# Supplementary figures and images for: Resistance diagnostics as a public health tool to combat antibiotic resistance: A model-based evaluation
Source: PLoS Biol. 2019 May 16;17(5):e3000250. doi: 10.1371/journal.pbio.3000250 (PMC6522007; doi:10.1371/journal.pbio.3000250)

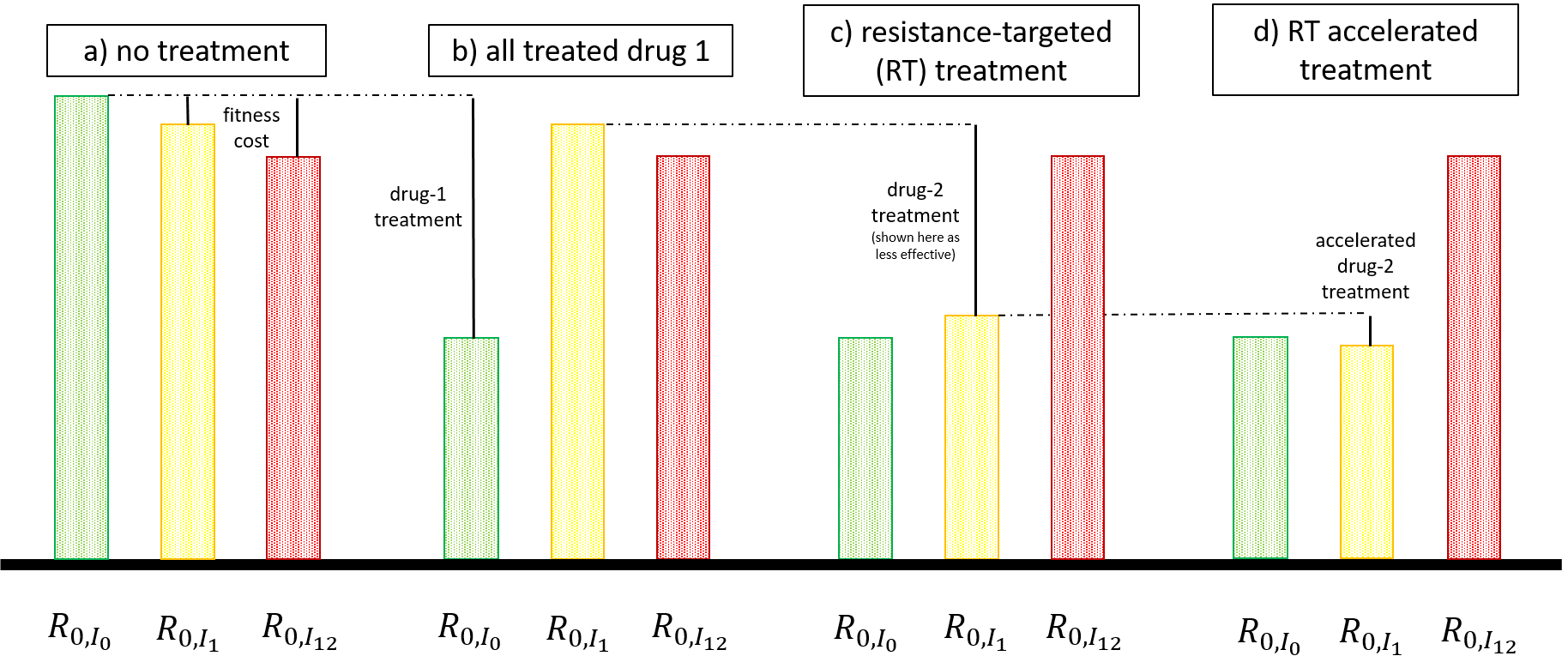

Supplement: S1 Fig — Reproduction numbers of sensitive strain (I0), drug 1–resistant strain (I1), and pan-resistant strain (I12), under various medical interventions: (a) no treatment, I0 enjoys advantage due to fitness costs associated with resistance; (b) all treated with drug 1, I1 and I12 enjoy advantage due to drug 1 resistance; (c) resistance-targeted treatment, I1 now at disadvantage unless (as shown) drug 2 is sufficiently less effective than drug 1; and (d) resistance-targeted accelerated treatment, whereby strain-I1 infections come more quickly to medical attention due to heightened discovery. (TIF) [file pbio.3000250.s002.tif]

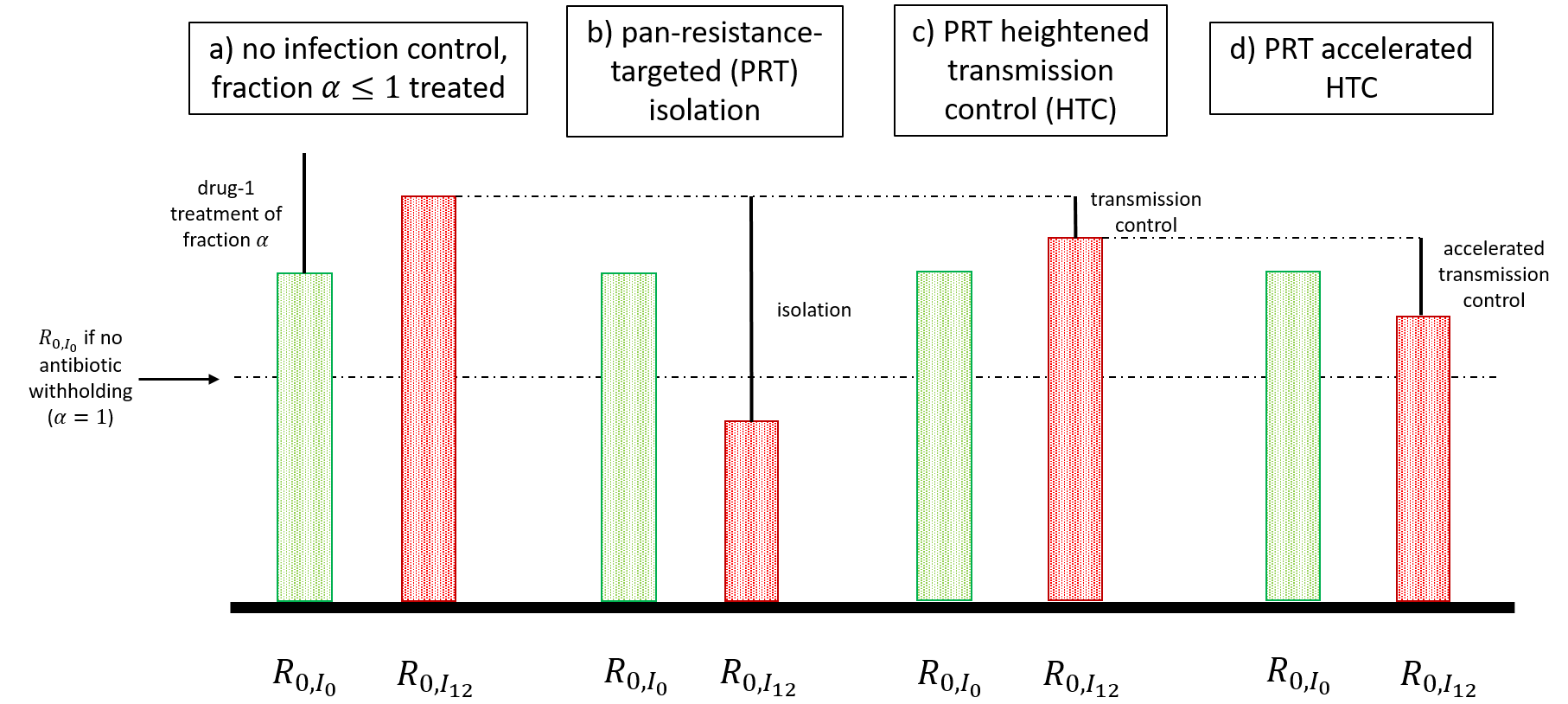

Supplement: S2 Fig — Reproduction numbers of sensitive strain (I0) and pan-resistant strain (I12), under various medical interventions: (a) fraction α≤1 treated with drug 1, I12 enjoys advantage due to surviving treatment unless α is sufficiently small; (b) PRT isolation, I12 now at disadvantage even if all sensitive infections treated with drug 1; (c) PRT HTC, with HTC shown here as much less effective than isolation; (d) PRT accelerated HTC, whereby strain-I12 infections come more quickly under control due to heightened discovery. HTC, heightened transmission control; PRT, pan-resistance targeted. (TIF) [file pbio.3000250.s003.tif]

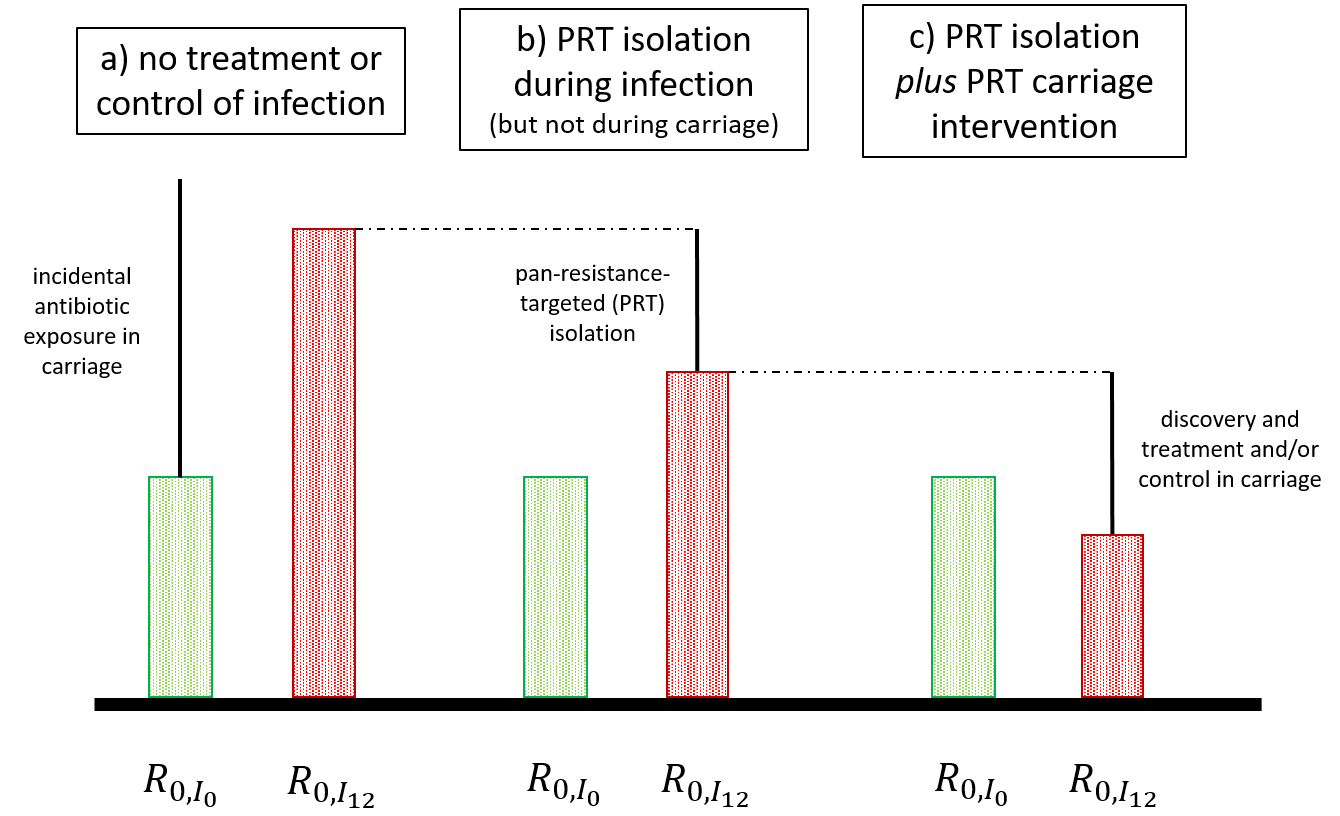

Supplement: S3 Fig — Reproduction numbers of sensitive strain (I0) and pan-resistant strain (I12) of an opportunistic pathogen, under various medical interventions: (a) no treatment or control, I12 enjoys advantage due to surviving incidental antibiotic exposure; (b) PRT isolation during infection, I12 may still enjoy advantage if the pathogen dwells mainly in carriage; (c) PRT isolation during infection plus PRT carriage intervention, I12 now at overall disadvantage so long as carriage intervention more effective at clearing pan-resistant bacteria than incidental exposure is at clearing sensitive bacteria (see Part C for details). PRT, pan-resistance targeted. (TIF) [file pbio.3000250.s004.tif]

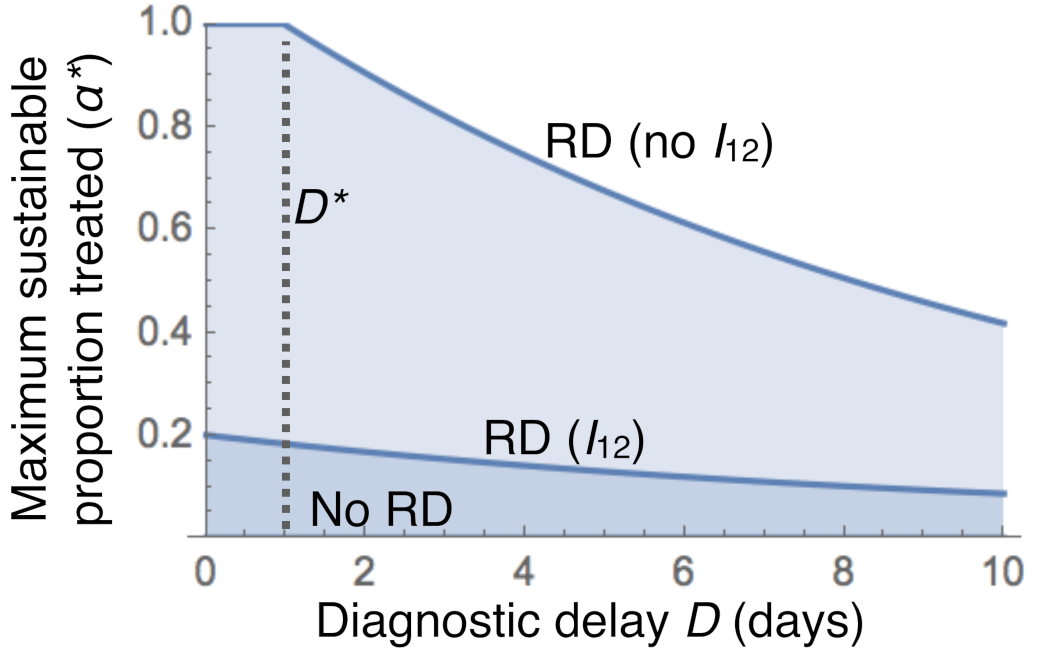

Supplement: S4 Fig — The maximal proportion of sensitive infections that can be treated without causing an increase in resistance (α*) is plotted against diagnostic delay D, assuming that all infections are immediately discovered and there are no biological fitness costs. The dashed vertical line indicates the longest diagnostic delay (D*) consistent with selection against drug 1 resistance while treating all cases. Three scenarios are shown: RD not available (No RD), for which α* = 0; RD available with delay and pan-resistance not yet emerged (RD, no I12); RD available with delay and pan-resistance widespread (RD, I12). Parameters (rates per day): γ0I=0.1, γ1I=γ2I=0.2,βI=0.2. RD, resistance diagnostics. (TIF) [file pbio.3000250.s005.tif]

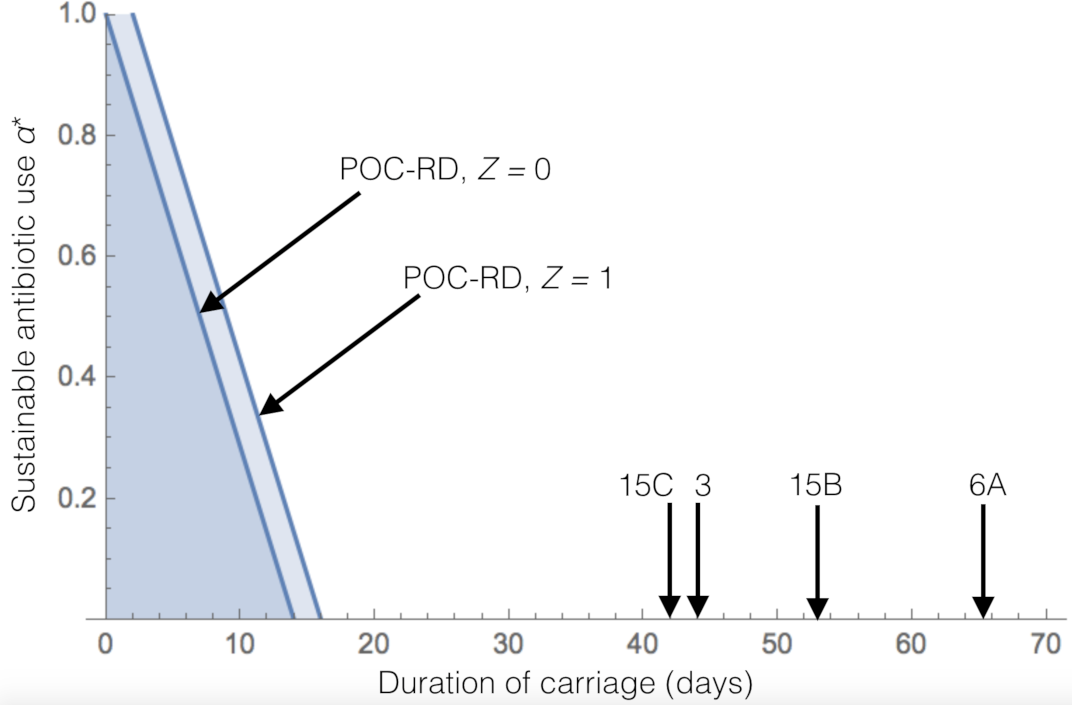

Supplement: S5 Fig — The maximal proportion of sensitive infections that can be treated without causing an increase in drug 1 resistance (α*) is plotted against the expected duration of carriage. Two POC-RD scenarios are shown: with (Z1I=0) and without (Z1I=1) transmission control. Vertical arrows represent pneumococcal serotypes with below-average carriage duration (see main text Fig 4 for broader range of serotype carriage durations). The remaining parameters (rates per day) are d = 0.001, ϕ1C = 5 × 10^−4^, ϕ2C = 0, γ1I=γ2I = 1, γ0I = 0.125. We make the simplifying assumption that baseline carriage and infection transmission rates are identical (βC = βI = β), ensuring that α* does not depend on β. Details on parameterization are in Part G. POC-RD, point-of-care resistance diagnostics. (TIF) [file pbio.3000250.s006.tif]
